# Developing interventions to improve detection of depression in primary healthcare settings in rural Ethiopia

**DOI:** 10.1192/bjo.2024.1

**Published:** 2024-02-26

**Authors:** Mekdes Demissie, Rahel Birhane, Charlotte Hanlon, Tigist Eshetu, Girmay Medhin, Abebaw Minaye, Kassahun Habtamu, Anthony J. Cleare, Barkot Milkias, Martin Prince, Abebaw Fekadu

**Affiliations:** Centre for Innovative Drug Development and Therapeutic Trials for Africa (CDT-Africa), Addis Ababa University, Ethiopia; and School Of Nursing and Midwifery, College of Health Sciences and Medicine, Haramaya University, Ethiopia; Centre for Innovative Drug Development and Therapeutic Trials for Africa (CDT-Africa), Addis Ababa University, Ethiopia; Department of Psychiatry, College of Health Sciences, Addis Ababa University, Ethiopia; and Centre for Global Mental Health & Centre for Implementation Science, Health Service and Population Research Department, Institute of Psychiatry, Psychology and Neuroscience, King's College London, UK; Aklilu Lemma Institute of Pathobiology, Addis Ababa University, Ethiopia; School of Psychology, College of Education and Behavioral Studies, Addis Ababa University, Ethiopia; Center for Affective Disorders, Institute of Psychiatry, Psychology and Neuroscience, King's College London, UK; Department of Psychiatry, College of Health Sciences, Addis Ababa University, Ethiopia; Centre for Global Mental Health, Health Service and Population Research Department, Institute of Psychiatry, Psychology and Neuroscience, King's College London, UK; and King's Global Health Institute, Faculty of Life Sciences and Medicine, King's College London, UK; Centre for Innovative Drug Development and Therapeutic Trials for Africa (CDT-Africa), Addis Ababa University, Ethiopia; Department of Psychiatry, College of Health Sciences, Addis Ababa University, Ethiopia; Center for Affective Disorders, Institute of Psychiatry, Psychology and Neuroscience, King's College London, UK; and Department of Global Health & Infection, Brighton and Sussex Medical School, UK

**Keywords:** Depression, case identification, depression recognition, intervention, detection of depression

## Abstract

**Background:**

The poor detection of depression in primary healthcare (PHC) in low- and middle-income countries continues to threaten the plan to scale up mental healthcare coverage.

**Aims:**

To describe the process followed to develop an intervention package to improve detection of depression in PHC settings in rural Ethiopia.

**Method:**

The study was conducted in Sodo, a rural district in south Ethiopia. The Medical Research Council's framework for the development of complex interventions was followed. Qualitative interviews, observations of provider–patient communication, intervention development workshops and pre-testing of the screening component of the intervention were conducted to develop the intervention.

**Results:**

A multicomponent intervention package was developed, which included (a) manual-based training of PHC workers for 10 days, adapted from the World Health Organization's Mental Health Gap Action Programme Intervention Guide, with emphasis on depression, locally identified depressive symptoms, communication skills, training by people with lived experience and active learning methods; (b) screening for culturally salient manifestations of depression, using a four-item tool; (c) raising awareness among people attending out-patient clinics about depression, using information leaflets and health education; and (d) system-level interventions, such as supportive supervision, use of posters at health facilities and a decision support mobile app.

**Conclusions:**

This contextualised, multicomponent intervention package may lead to meaningful impact on the detection of depression in PHC in rural Ethiopia and similar settings. The intervention will be pilot tested for feasibility, acceptability and effectiveness before its wider implementation.

Depression affects 10–20% of the world's population,^1–3^ with even higher prevalence among primary healthcare (PHC) attendees.^4–6^ In 2019, depression was the sixth most disabling condition globally among people aged 10–49 years,^7^ and is projected to be the second leading contributor to disability-adjusted life-years by 2030.^8^ In Ethiopia, depression was the second leading cause of years lived with disability in 2019.^9^ However, detection of depression in the PHC setting is low,^4,10^ with more than 90% of the cases undetected in low- and middle-income countries (LMICs).^4^ Undetected depression is associated with unfavourable clinical and functional outcomes,^11,12^ suicidality^13^ and increased health service utilisation.^14^ In response, the World Health Organization (WHO) developed the Mental Health Gap Action Programme Intervention Guide (mhGAP-IG) as a training tool to support integration of mental healthcare in LMICs,^15–17^ including Ethiopia. However, the detection of depression by the trained PHC workers remained very low.^18^

## Formative studies

In recognition of this challenge, the Improving Detection of Depression in Primary Care in Sub-Saharan Africa Study (IDEAS) was designed.^19^ The project attempted to first understand the nature of depression in the local setting through extensive qualitative studies,^20^ and review of the conceptualisation of depression in the region followed by a review of the interventions developed to improve detection of depression.^21,22^ The study also evaluated the validity of several screening tools that could be used to enhance the detection of depression.^23^ In the qualitative study, we identified ‘noise intolerance’ and ‘irritability’ to be the most frequently and spontaneously reported symptoms of depression.^20^ A systematic review of 25 studies from sub-Saharan Africa that assessed the conceptualisation of depression indicated that depression was an important condition rooted in social adversity. It was characterised by negative affect, sadness, somatic symptoms and even typical DSM symptoms.^21^ The study emphasised the need for any intervention development to take into account the target population's culture and social context.^21^ A systematic review of 58 studies showed that interventions such as clinician training, screening, feedback and guidelines need to be used in combination, to increase recognition of depression in the PHC setting.^22^ We evaluated the psychometric properties of different screening instruments, including four versions of the Patient Health Questionnaire (PHQ) – PHQ-9, PHQ-2 and PHQ-11 (PHQ-9 plus the two new items of noise intolerance and irritability), and PHQ-15 – and WHO-5 well-being scale. While all the scales were highly correlated, the PHQ-15 had the weakest association with the diagnosis of depression. Within the PHQ-11, the top three items that were strongly associated with diagnosis of depression, in descending order, were irritability (odds ratio (OR) 2.64; 95% CI 1.47–4.72); feeling down, depressed or hopeless (OR 2.46; 95% CI 1.87–3.23); and noise intolerance (OR 2.25; 95% CI 1.30–3.91). Little interest or pleasure in doing things was the least associated item with diagnosis (1.30; 1.05–1.61).^23^

Overall, the formative work indicated that multifaceted and multilevel interventions were needed to enhance detection.^19–25^ Therefore, this study aimed to report the process used to develop a contextualised intervention package to increase identification of depression by PHC workers in Ethiopia.

## Method

### Design and context

**Fig. 1:**
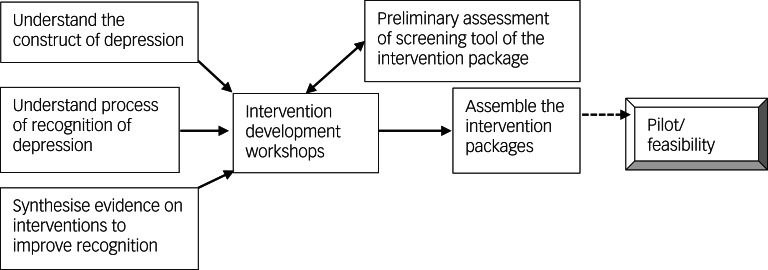
Overview of the process of development of the intervention packages.

The development of the intervention package followed two phases: inception (formative) and intervention development. Details of the findings from the inception phase were published previously,^20–23^ and their contributions to the intervention development are summarised in Table 3. The intervention development phase built from the formative phase and involved a qualitative study, structured observation of provider–patient communication, a series of intervention development workshops with relevant stakeholders and preliminary assessment of the implementation of the screening intervention (Fig. 1). The study was conducted between 2018 and 2021.

### Setting

The study was conducted in the Sodo district of the Gurage Zone in south central Ethiopia. The district is located 100 km from the capital, Addis Ababa, and had an estimated total population of 173 185 in 2014.^26^ Sodo district has eight health centres and one primary hospital. Four health centres (Adele, Tiya, Kela and Wulawula) and Bui primary hospital were included in this study. The PHC workers in the district have been previously trained with the WHO's mhGAP intervention guide to provide care for priority mental health conditions through the Programme for Improving Mental Healthcare (PRIME) project (2011–2018).^27^

### Participants and data collection methods

#### Qualitative

To understand the barriers to, and facilitators of, detection of depression, we conducted three focus group discussions and in-depth interviews with PHC workers (*n* = 2), and in-depth interviews with people who were diagnosed with depression (*n* = 28; 16 females).

#### Provider–patient communication (structured observation of the process of recognition)

We conducted structured observations of provider–patient communication by PHC workers to understand patient–clinician interactions. A total of 30 consecutive PHC consultations in two PHC facilities with mhGAP-IG-trained staff were observed. Each consultation was rated by trained psychiatric nurses with the 37-item Calgary–Cambridge Guide (C-CG).^28^ The scale has five domains: initiating the session (seven items), gathering information (11 items), providing structure (four items), building relationships (ten items) and closing the session (five items). Each item was rated as not done/poor (0), adequately done (1), good (2) or not applicable (4). A trained psychiatric nurse completed the scale.

#### Intervention development workshops

The workshops discussed findings from the inception phase and obtained feedback on a draft intervention package to improve detection of depression in PHC. We conducted four separate workshops with (a) the community advisory board, (b) Sodo district health office representatives and PHC workers, (c) IDEAS project investigators and (d) mental health experts and researchers. The last two groups were involved in developing the actual intervention package based on the formative works, whereas the first two groups commented on the draft package. Participants were selected purposively based on their experience in intervention development, their position in the district health office or experience providing mental health services in the study setting. The workshops with experts and researchers, as well as the IDEAS research team, were held in Addis Ababa, whereas the workshops with the community advisory board and district health office representatives were held in Sodo district. A.F. and R.B. facilitated the sessions. Two of the workshops were held for half a day and one was held for a full day. Each workshop had two sections. First, we presented findings from the inception phase, and then participants were invited to discuss the findings and suggest options to improve the detection of depression within their working context. Finally, the facilitators summarised the discussion, recorded key points and the participants’ consensus on components included in the intervention manual on a flip chart, and requested participants confirmation that the key recommendations were captured. For each workshop, minutes were taken and they were audio recorded.

#### A preliminary assessment of a screening component for the intervention package

The assessment was conducted in four PHC centres to decide on the items that would be included and the response categories. Two potential candidate scales were the standard PHQ-2 and a new scale that included the PHQ-2 plus two contextually salient items (noise intolerance and irritability), using either the standard PHQ rating system or simple’ yes/no’ response categories. Ease and length of administration, as well as performance, were investigated.

### Analysis

In the qualitative studies, we focused on the identification of the facilitators and barriers for detection of depression by thematic analysis. First, the in-depth interviews and focus group discussions were transcribed verbatim and imported into Open Code for Windows, version 4.03 (Umea University, Sweden; see https://www.umu.se/en/department-of-epidemiology-and-global-health/research/open-code2/). Two authors (R.B. and T.E.) did a line-by-line coding of two randomly selected transcripts, discussed the codes and developed a codebook. Then, the remaining transcripts were coded based on the codebook, and added and defined new codes when necessary. Then, the similar codes were grouped into clusters to capture the essence of particular themes. Simple descriptive statistics was employed to summarize observations of the 30 clinical encounters from the C-CG and understand the provider–patient communication. For the four intervention development workshops, proceedings and consensus were prepared from the flipcharts we used to write the key notes and participants’ consensus on components, the minutes of the meetings and audio recordings.

### Quality of reporting

We used the 14-item Guidance for Reporting Intervention Development (GUIDED) checklist (Supplementary File 1 available at https://doi.org/10.1192/bjo.2024.1) to improve the quality of reporting of intervention development.^29^

### Ethics approval and consent to participate

The authors declare that all procedures contributing to this work comply with the ethical standards of the relevant national and institutional committees on human experimentation and with the Helsinki Declaration of 1975, as revised in 2008. All procedures involving human patients were approved by the Addis Ababa University Institutional Review Board (reference number 007/18/Psy (2018-2021)) and the study was registered (trial ID: PACTR202206723109626). Written informed consent was obtained from each participant. Non-literate participants gave fingerprints to signify their willingness to participate, and this was witnessed by an independent person.

## Results

### Barriers to and facilitators for detection of depression

Participants reported three types of barriers: illness- and patient-related factors, health worker-related factors and health system-related factors. For health workers, inadequate knowledge and skills to identify depression symptoms, lack of attention given to depression and inability to explore a patient's psychosocial conditions that could signal depression were reported as barriers. Regarding health system-related factors, participants identified high patient flow and lack of necessary infrastructure (like laboratory equipment at the health facility) as reasons for inadequate exploration of patient information and physical examination. Regarding illness-related factors, predominance of somatic over emotional symptoms was highlighted as a challenge to the detection of depression. In addition, PHC workers emphasised that patients do not disclose their psychosocial issues and mostly present with comorbid medical conditions. As a result, patients are misdiagnosed and treated as having a medical condition, and visit the health facility frequently. Most PHC workers mentioned screening as a facilitator for identifying patients with depression. However, workload and inadequate time to administer the screening tool were considered as potential challenges. Thus, they suggested a short screening tool that can be used at the triage to reduce workload at the out-patient department. Patients also indicated that they were diagnosed as having depression for the first time after they were asked about depressive symptoms through an interview scale during the PRIME study. The mhGAP training was the other factor highlighted as a facilitator, although some PHC workers mentioned that the content, and time allocated during the training on how to assess depression, was inadequate.

### Provider–patient communication

On average, consultations took 17.7 (s.d. = 4.2) minutes. None of the 30 observed consultations were rated as good in any domain of the C-CG. In the initiating domain, the PHC workers’ identification of reasons for consultation was rated as adequate, although their initial rapport was rated poor. The information gathering domain was rated as adequate, except for ‘asking easily understood questions’, ‘summarising what the patients said to verify understanding the problem’ and ‘exploring the patient's perspective’. The third domain assessing PHC workers’ organisation of history taking was rated as poor. In ‘building relationship’, using non-verbal cues and providing support were adequate. However, patients’ involvement in their assessment was poor. The closing domain was also rated poor in the majority of the consultations (Supplementary File 2).

### Intervention development workshops

Four separate workshops were organised with a community advisory board for the study (*n* = 19, 5 female and 12 male), mental health experts and researchers (*n* = 8, 4 male and 4 female), IDEAS investigators (*n* = 6, 4 male and 2 female) and district level health office representatives and PHC workers (*n* = 15, 11 male and 4 female) (Table 1).

**Table 1:** Intervention development workshop participants

Stakeholder group	Number of participants
Community advisory board (*N* = 19)	
People with mental illness	2
Caregivers	2
Community leaders (security, gender office, women and youth affairs, religious affairs and education)	12
Sodo district health office administrators	3
Workshop with mental health experts and researchers	8
Workshop with IDEAS investigators	6
Workshop with district health office representatives and primary healthcare workers (*N* = 15)	
Sodo district health office	4
Primary hospital	3
Health centres	8

IDEAS, Improving Detection of Depression in Primary Care in Sub-Saharan Africa Study.

#### Community advisory board meeting

The community advisory board members perceived that the identification of depression was low. They also suggested that depression not being considered as an illness by the community was an important reason for the underrecognition, and that increasing community awareness either when patients visited the health facilities or in the villages was important. They also recommended separate training of the PHC workers on depression.

#### Workshop with mental health experts and researchers

Participants suggested two types of interventions: screening with feedback and training for PHC workers. Screening with feedback was suggested based on the evidence from the systematic review and PHC worker recommendations in the qualitative study. To decide on which specific screening instrument to use, participants suggested a preliminary assessment of four brief screening tools in four different health facilities that have mhGAP-trained staff: (a) PHQ-2 with a simple yes/no option, (b) PHQ-2 with standard PHQ response categories, (c) PHQ-2 plus two additional items (irritability and noise intolerance) with yes/no response categories and (d) PHQ-2 plus the two additional items with standard response categories. Participants believed the PHQ-2 to be the most feasible, given its brevity. The additional two symptoms identified from the qualitative study (irritability plus noise intolerance) were also highly associated with clinician diagnosis of depression, suggesting their usefulness in screening for depression.

The expert group also suggested the cut-off score for the various PHQ options for screen positivity. Thus, for the standard rating approach, a total score of 3 or more was considered compatible with screen-positive status. For the PHQ options with yes/no response categories, a ‘yes’ response to any of the items in the tools was considered compatible with screen-positive status. Moreover, the participants suggested the screening tool should be administered in the triage office by triage nurses, and to refer those who screened positive to mhGAP-trained PHC staff with the result of the screening.

Regarding the training, participants suggested practice-based PHC worker training for 2 weeks, consisting of theoretical (1 week) and practical (1 week) training. The emphasis was on skills and recommended integration of skills and patient trainers even within the theoretical training. The participants emphasised communication skills in response to the findings of the C-CG. The theoretical training was also further split into two, with 3 days dedicated primarily to depression and 1 days dedicated to the broader issue of assessment and other mental disorders that are relevant to depression, such as adjustment disorder, bipolar disorder, substance misuse and suicidality.

#### Workshop with the IDEAS project team

During a workshop with the IDEAS research team, the participants discussed the findings of the inception phase, the first workshop and the preliminary assessment of the screening tools. Based on the findings, they recommended the use of the four-item tool (PHQ-2 plus two additional items) with a yes/no response category as a screening tool, with feedback at the triage facility. They suggested adapting the mhGAP intervention guide for depression. Moreover, they suggested that the manual include essential care practice with a focus on principles of good communication and building skills for assessment of priority problems. They also suggested training with the adapted tool, and system-level intervention components such as supportive supervision, a weekly quality improvement review meeting and the preparation of a poster. Additionally, they suggested health education in the out-patient clinics and distribution of information leaflets to improve awareness about depression. Finally, they suggested that, although the primary outcome of the intervention is improving detection of depression, an economic evaluation and other process measures, such as fidelity, feasibility and acceptability of the intervention package, change in PHC workers’ skills and attitude toward depression, needed to be assessed.

#### Workshop with district health office representatives and PHC workers

In the final workshop with the district health office representatives and PHC workers, findings on the formative study, systematic review and previous workshops were presented. Then, the participants discussed the feasibility and acceptability of potential intervention components and areas that needed further adaptation. All the participants mentioned that the suggested intervention components are important and appeared feasible to implement. In addition to the already suggested intervention components, participants recommended a mobile app to support clinical decisions and regular health education during waiting time. They suggested intervention packages should be tested at three intensity levels: (a) test the core intervention components (training, supportive supervision, mobile app to support clinical decision, weekly quality improvement review meeting and poster) in one facility (level 1 intensity), (b) test the core intervention (level 1 intensity) plus screening and feedback (level 2 intensity) and (c) test level 2 intensity plus awareness raising through health education and the information leaflet (level 3 intensity). They recommended using a high-functioning facility with staff trained in the standard mhGAP intervention as a comparison facility.

### Preliminary assessment of the screening tool

As shown in Table 2, a total of 271 patients (55% female) were screened across four health facilities. The majority of patients (92.2%) reported that they were comfortable with being asked about their emotions and that the questions were easy to understand (85.2%). The average time taken to administer the screening instruments ranged from 0.59 min (PHQ-2, yes/no responses) to 1.83 min (PHQ-2 plus two additional items, standard responses). In the contextually adapted screener with yes/no responses, 69 out of 139 participants (49.6%) screened positive, with 14.5% (10/ 69) diagnosed by PHC staff as having depression. This declined to 13 out of 75 (17.3%) participants with the standard response categories, with two of the 13 (15.4%) identified by PHC workers as having depression. In the conventional PHQ-2 screener (with standard or yes/no responses), none of the participants who screened positive were diagnosed as having depression by PHC workers. Patients, triage staff and heads of health facilities reported that the administration of the screening tools was feasible and acceptable. The triage staff reported that it was not disruptive to their work, and that that it was concordant with their other screening activities. Although the scoring was generally straightforward, triage staff preferred the simple yes/no category option because of ease of administration.

**Table 2:** Preliminary assessment of the four different screening tools

Screening tool	Response category	Average time taken to administer in minutes (range)	Total PHC attendees screened	Screened positive	Diagnosed by PHC workers among those who screened positive
PHQ-2	Yes/no	0.6 (0.25–2.5)	33	18	0
PHQ-2	PHQ-2 standard responses	1.2 (0.5–2.3)	24	8	0
PHQ-2 plus two cultural items	Yes/no	0.8 (0.5–4.6)	139	69	10
PHQ-2 plus two cultural items	PHQ-2 standard responses	1.8 (0.4–3.4)	75	13	2

PHC, primary healthcare; PHQ, Patient Health Questionnaire.

### Details of the intervention packages developed

All of the formative findings were integrated into the intervention packages (Table 3), and intervention components were designed to be delivered at various levels of the health system (Table 4).

**Table 3:** Contribution of the specific inception phase activities to the development of the intervention package to improve detection of depression in primary healthcare in Ethiopia

Methods	Findings	Contribution to the development of the intervention components
Qualitative and psychometric studies	Symptoms such as noise intolerance and irritability were frequently and spontaneously reported by the patients Low awareness about depression in the community The total scores generated from the PHQ-2, PHQ-9 and PHQ-11 were highly correlated	Local expressions included for communication and information leaflets Identifying symptoms relevant to the study context The need for awareness raising identified Candidate screening instruments identified
Understanding the process of recognition of depression in PHC	Barriers for detection Inadequate ‘knowledge and skills’ of professionals about assessment of depression Patients presenting with vague depressive symptoms or more somatic than emotional symptoms and comorbidity Lack of open discussion between patient and PHC workers Inadequate content and length of training on depression assessment Facilitators for detection Using screening tool Provision of mhGAP training Provider–patient communication Patient–provider communication was inadequate; training needed for providers	A more focused theoretical and practical training on depression Adequate time was given to the training (10 days) to ensure that it is well absorbed by the trainees Case stories were considered mandatory during the training Make training sessions more interactive by including role plays to improve the clinical and communication skills To include real case demonstrations to improve providers’ skills To consider screening in the triage
Systematic review	Single intervention alone less likely to work Combinations deemed useful were education, screening and feedback Implementation of quality improvement programmes were effective to improve detection of depression	Screening with feedback, training of PHC workers and supervision were included in the intervention package Quality improvement programme was incorporated in the intervention package
Workshops	Recommendations on potentially useful intervention components Screening tool with training Mobile app that supports clinical decision Adaptation of the mhGAP intervention guide Information leaflet Posters and quality improvement/review meeting	Intervention components proposed Preliminary assessment and piloting sites, evaluation methods were suggested Awareness raising activities proposed to be included in the intervention package Facilitator guide developed Mobile apps, poster and leaflets developed
Preliminary assessment	The tested screening instruments were feasible and acceptable to be used at the triage Four items with yes/no response categories showed good ability to identify people with depression	Four items screening instrument was recommended for use at the triage for screening purposes (depressed mood, loss of interest, irritability and noise intolerance Yes/no response option perceived easy to administer

PHQ, Patient Health Questionnaire; PHC, primary healthcare; mhGAP, Mental Health Gap Action Programme.

**Table 4:** Summary of intervention components developed to improve detection of depression in a primary healthcare setting

Intervention level	Interventions
Health system	Supportive supervision, mobile app to support clinical decision, poster and quality improvement meetings
Facility	Screening at triage with an adapted tool and feedback
Primary healthcare workers	Ten days of training, consisting of theoretical and practical sessions; pocket guide
Community	Health education and information leaflet

The training manual had two components: a depression care trainer's manual and facilitator's guide, and a depression care booklet. These were adapted from the WHO's mhGAP-IG, with more emphasis on principles of good communication and building skills for assessment of priority problems and providing basic psychosocial interventions (e.g. self-care, reducing stress, problem-solving). In addition, the number of modules was reduced to essential care practice, depression, self-harm/suicide and other significant mental health complaints.

In each of the modules the following, were changed. Optional person stories in the mhGAP-IG were made mandatory. More activities such as role-plays were added, with the aim of building the clinical skills of trainees. Two people with lived experience of depression were involved as trainers, to share their lived experience of the depression treatment pathways. This was to support the trainees’ assessment and management skills and increase empathy. In the depression module, local findings of common presenting symptoms of depression, specifically irritability and noise intolerance, were incorporated as core symptoms in the assessment algorithm. Likewise, common symptoms of psychosis were included in the depression algorithm so that trainees would be able to identify depression with psychotic symptoms and distinguish between depression and other psychotic disorders. More emphasis was also given to identification of history of mania and diagnosing bipolar depression. In the pharmacological management section, emphasis was given to prescribing antidepressants for depression with psychotic symptoms. The manual was prepared for a 2-week training course and was delivered by psychiatrists (1 week theoretical and 1 week practical). The practical session was to include assessments of routine out-patient attendees and those with confirmed depression. Additionally, the C-CG was included to assess and provide feedback on PHC workers’ communication skills during the training. The training was designed to be interactive by adding role-plays and video demonstrations.

#### Poster

A poster to be displayed in the out-patient clinic was developed. The poster described common presentations of priority mental health conditions in the mhGAP intervention guide.

#### Mobile app

The mobile app was developed to assist clinical decisions. The app is named the Depression Care app. The app was developed with mhGAP materials and primary data from the IDEAS project. The first section guides the PHC worker in assessment of depression, and the remaining sections provide guidance on the management of depression. The management section includes pharmacological interventions; psychoeducation including stress management, promoting functioning and teaching relaxation techniques; and a section with instructions to be considered during follow-up visits.

#### Screening intervention

Based on the findings of the preliminary assessment, it was decided to use the culturally adapted four-item screening tool (the PHQ-2 and the two additional items) with yes/no response categories. Hence, the four items in the screening instrument were depressed mood, lack of interest, irritability and noise intolerance. Any positive response was considered as a positive screen for possible depression. The screening instrument was to be administered by triage nurses, and the results were to be given to PHC workers that were trained to provide the intervention.

#### Information leaflet and health education

Two types of leaflets were prepared: one specific to depression and the other focused on mental illness in general. The depression leaflet included information about depression and its symptoms, causes, management options and self-care. PHC workers were also to provide health education to PHC attendees about depression.

#### Quality improvement meeting

This was designed to be held weekly for PHC workers and health facility heads, to discuss the progress of the implementation of the intervention package and its challenges.

## Discussion

To our knowledge, this is one of the most comprehensive intervention studies focusing on improving detection of depression in an LMIC. Although a few studies have evaluated such interventions, no robust evidence exists on effective interventions from LMICs.^4^ A study that indicated benefit from a 5-day comprehensive training package has not been replicated.^30,31^ The IDEAS multicomponent interventions were co-developed with rich contextual information that revisited the nature of depression and how it is conceptualised and communicated, with a focus on addressing factors that were considered barriers to recognition of depression, such as low practitioner expertise, high patient flow, low awareness of patients about depression, the need for locally specific expressions of symptoms and health system challenges, such as infrastructure and system support.

### Adapting a training package

Although the training package was based on the mhGAP-IG, major modifications were made in content, delivery and duration. One of the major innovations was the involvement of people with lived experience of depression in the training. This was in line with studies that acknowledged the role of people with lived experience in teaching based on their experience,^32^ which also makes it easier to link the course content with practice.^33^ The current depression intervention package was designed to be delivered over 10 days, with 5 days of theoretical and 5 days of practical training, with ample opportunities to conduct supervised interviews. This is in contrast to the standard mhGAP training, which allots 5 days to cover all priority mental health conditions without extended practical training.^34^ The IDEAS training approach had a clear impact on the confidence as well as practice of healthcare providers.

### Mobile app

The mobile app was included to support clinical decision-making. Findings from previous systematic reviews showed that the use of mobile technology improved diagnosis and treatment decisions,^35^ and the approach was also perceived positively by PHC workers.^36^ Unlike the application of the WHO mhGAP-IG,^37^ the IDEAS mobile app included irritability and noise intolerance as core depressive symptoms in the assessment algorithm, enabled exploration and exclusion of psychotic symptoms, and gave opportunity to practitioners to ask about imminent suicide risk/self-harm if the patient reported depressive symptoms. These modifications distinguished the mobile app from the WHO mhGAP mobile app, available as an electronic version of the mhGAP-IG.^37^

### Screening

Routine screening for depression is recommended as one strategy for improving detection of depression.^38^ Previous studies have reported improved detection of depression,^39–42^ particularly if screening was accompanied by feedback.^43^ Because of concerns about time constraints and other resources, potential for overreporting and work burden,^44,45^ screening was the last addition to the intervention package. However, the high patient flow, low exploration of depressive symptoms by professionals and challenge that patients rarely reported psychosocial problems identified in the formative study compelled the team to explore the potential benefit of screening. A four-item screening tool (PHQ-2 plus two additional items), with contextually relevant symptoms^20,46^ and a simple administration mechanism, was devised and tested. The preliminary assessment results in the current study confirmed the feasibility, acceptability and potential utility of using this screening tool. Although many depression screening tools have been validated against gold-standard clinical measures in PHC contexts in sub-Saharan Africa, we are not aware of any studies that have investigated how screening can be implemented in routine settings. The new symptoms also appear to be easy to administer, with acceptable utility. However, the high rate of screen positivity reduces the use of the screening tool in routine care. Further development work is required.

### Enhancing patient awareness

The focus on improving patient awareness as part of the intervention packages was informed by the perceived need in the formative study, and was in line with previous studies that reported improvement in help-seeking behaviour for mental illnesses following such intervention.^47^ The leaflets appear to have good acceptability and utility. However, their primary utility lies in being employed as part of a fuller treatment package.

### Limitations

The study has some limitations. First, although people with depression were involved in qualitative studies and in the community advisory board meeting, their primary role was in supporting the intervention delivery instead of the intervention development process. Second, the intervention was developed for use in predominantly rural districts in south Ethiopia. Additional adaptations may be required for use in other settings, which should be part of new evaluation studies. Although the screening tool is likely to be useful, the risk of overdiagnosis and potential harms related to false positives require further evaluation. Although medication was purchased through the project, sustainable support is required to ensure ongoing availability of medicines where these are indicated. Other structural challenges, such as clinic spaces, were beyond the scope of the study. The study team was only able to play an advocacy role. Finally, the training was provided by psychiatrists, which may affect generalisability.

In conclusion, we have co-developed a multicomponent intervention for improving detection of depression in a PHC setting in sub-Saharan Africa. The development process considered locally meaningful conceptualisations and representations of depression, and was informed by interventions tested globally in PHC settings. We are not aware of others studies that have taken such a comprehensive approach. The intervention will be pilot tested for feasibility, acceptability and effectiveness before its wider implementation. If the package is proven to be effective, it will offer significant hope for scaling up mental healthcare through PHC integration.

## Supporting information

Demissie et al. supplementary material Demissie et al. supplementary material

## Data Availability

The data that support the findings of this study are available from the corresponding author, A.F., on reasonable request..
