# Supplementary material for: Developing interventions to improve detection of depression in primary healthcare settings in rural Ethiopia
Source: BJPsych Open. 2024 Feb 26;10(2):e52. doi: 10.1192/bjo.2024.1 (PMC10897685; doi:10.1192/bjo.2024.1)
Supplement: Demissie et al. supplementary material [file S2056472424000012sup001.docx]

Supplementary file 1

**GUIDED – a guideline for intervention development studies.**

| Item description | Explanation | Page in manuscript where item is located | Other* |
| --- | --- | --- | --- |
| 1. Report the context for which the intervention was developed. | Understanding the context in which an intervention was developed informs readers about the suitability and transferability of the intervention to the context in which they are considering evaluating, adapting or using the intervention. Context here can include place, organisational and wider socio-political factors that may influence the development and/or delivery of the intervention. | Page 5 |  |
| 2. Report the purpose of the intervention development process. | Clearly describing the purpose of the intervention specifies what it sets out to achieve. The purpose may be informed by research priorities, for example those identified in systematic reviews, evidence gaps set out in practice guidance such as The National Institute for Health and Care Excellence or specific prioritisation exercises such as those undertaken with patients and practitioners through the James Lind Alliance. | Page 3 and 4 |  |
| 3. Report the target population for the intervention development process. | The target population is the population that will potentially benefit from the intervention – this may include patients, clinicians, and/or members of the public. If the target population is clearly described then readers will be able to understand the relevance of the intervention to their own research or practice. Health inequalities, gender and ethnicity are features of the target population that may be relevant to intervention development processes. | Page 3 and 4 |  |
| 4. Report how any published intervention development approach contributed to the development process | Many formal intervention development approaches exist and are used to guide the intervention development process (e.g. six steps in quality intervention development (6SQuID) or The Person Based Approach to Intervention Development). Where a formal intervention development approach is used, it is helpful to describe the process that was followed, including any deviations. More general approaches to intervention development also exist and have been categorised as follows: Target Population-centred intervention development; evidence and theory-based intervention development; partnership intervention development; implementation-based intervention development; efficacy-based intervention development; step or phased-based intervention development; and intervention-specific intervention development. These approaches do not always have specific guidance that describe their use. Nevertheless, it is helpful to give a rich description of how any published approach was operationalised | Page 7 |  |
| 5. Report how evidence from different sources informed the intervention development process | Intervention development is often based on published evidence and/or primary data that have been collected to inform the intervention development process. It is useful to describe and reference all forms of evidence and data that have informed the development of the intervention because evidence bases can change rapidly, and to explain the manner in which the evidence and/or data was used. Understanding what evidence was and was not available at the time of intervention development can help readers to assess transferability to their current situation. | Page 15 |  |
| 6. Report how/if published theory informed the intervention development process. | Reporting whether and how theory informed the intervention development process aids the reader’s understanding of the theoretical rationale that underpins the intervention. Though not mentioned in the e-Delphi or consensus meeting, it became increasingly apparent through the development of our guidance that this theory item could relate to either existing published theory or programme theory. |  |  |
| 7. Report any use of components from an existing intervention in the current intervention development process. | Some interventions are developed with components that have been adopted from existing interventions. Clearly identifying components that have been adopted or adapted and acknowledging their original source helps the reader to understand and distinguish between the novel and adopted components of the new intervention. | Page 16 and 17 |  |
| 8. Report any guiding principles, people or factors that were prioritised when making decisions during the intervention development process. | Reporting any guiding principles that governed the development of the application helps the reader to understand the authors’ reasoning behind the decisions that were made. These could include the examples of particular populations who views are being considered when designing the intervention, the modality that is viewed as being most appropriate, design features considered important for the target population, or the potential for the intervention to be scaled up. | Page 11-13 |  |
| 9. Report how stakeholders contributed to the intervention development process. | Potential stakeholders can include patient and community representatives, local and national policy makers, health care providers and those paying for or commissioning health care. Each of these groups may influence the intervention development process in different ways. Specifying how differing groups of stakeholders contributed to the intervention development process helps the reader to understand how stakeholders were involved and the degree of influence they had on the overall process. Further detail on how to integrate stakeholder contributions within intervention reporting are available. | Page 11-13 |  |
| 10. Report how the intervention changed in content and format from the start of the intervention development process. | Due to the iterative nature of intervention development, the intervention that is defined at the end of the development process cab often be quite different from the one that was initially planned. Describing these changes and their rationale enhances understanding and enables other intervention developers to learn from this experience. For example, it may be that some intervention components were considered but ultimately discarded due to complexity or expense of delivery. |  | The prototype will be changed in accordance with the feasibility testing. |
| 11. Report any changes to interventions required or likely to be required for subgroups. | Specifying any changes that the intervention development team perceive are required for the intervention to be delivered or tailored to specific sub groups enables readers to understand the applicability of the intervention to their target population or context. These changes could include changes to personnel delivering the intervention, to the content of the intervention, or to the mode of delivery of the intervention. |  |  |
| 12. Report important uncertainties at the end of the intervention development process. | Intervention development is frequently an iterative process. The conclusion of the initial phase of intervention development does not necessarily mean that all uncertainties have been addressed. It is helpful to list remaining uncertainties such as the intervention intensity, mode of delivery, materials, procedures, or type of location that the intervention is most suitable for. This can guide other researchers to potential future areas of research and practitioners about uncertainties relevant to their healthcare context. | Page 20. |  |
| 13. Follow TIDieR guidance when describing the developed intervention. | Interventions have been poorly reported for a number of years. In response to this, internationally recognized guidance has been published to support the high quality reporting of health care, interventions and public health interventions. This guidance should therefore be followed when describing a developed intervention. | Page 16-17 |  |
| 14. Report the intervention development process in an open access format. | Unless reports of intervention development are available, people considering using an intervention cannot understand the process that was undertaken and make a judgment about its appropriateness to their context. It also limits cumulative learning about intervention development methodology and observed consequences at later evaluation, translation and implementation stages. Reporting intervention development in an open access (gold or green) publishing format increases the accessibility and visibility of intervention development research and makes it more likely to be read and used. Potential platforms for open access publication of intervention development include open access journal publications, freely accessible funder reports or a study web-page that details the intervention development process. |  | Done. |

*e.g. if item is reported elsewhere, then the location of this information can be stated here.

Citation: Duncan E, et al. BMJ Open 2020; 10:e033516. doi: 10.1136/bmjopen-2019-033516

**Supplementary File 2: Summary of consultation scores of PHC workers** **using the 37 item Calgary-Cambridge Guide (C-CG)**

|  |  | Good  Freq. (%) | Adequate  Freq. (%) | Not done/ Poor  Freq. (%) |  |
| --- | --- | --- | --- | --- | --- |
| **INITIATING THE SESSION** | | | | | |
| 1 | Greets patient | 0 (0) | 5 (16.67) | 25 (83.33) |  |
| 2 | Introduces self, role and nature of interview | 0 (0) | 0 (0) | 30 (100) |  |
| 3 | Demonstrates respect and interest, attends to patient’s physical comfort | 0 (0) | 0 (0) | 30 (100) |  |
|  | Overall score for establishing initial rapport | Mean: 0.167 SD: 0.38 min 0 max 1 | | | |
| 4 | Uses an appropriate opening question | 0 (0) | 30 (100) | 0 (0) |  |
| 5 | Listens attentively | 0 (0) | 28 (93.33) | 2 (6.67) |  |
| 6 | Confirms issues to be discussed/screens for further questions | 0 (0) | 28 (93.33) | 2 (6.67) |  |
| 7 | Negotiates agenda | 0 (0) | 30 (100) | 0 (0) |  |
|  | Overall score for Identifying the reason for the consultation | Mean: 3.867 SD: 0.51 min 2 max 4 | | | |
| GATHERING INFORMATION | | | | | |
| 8 | Encourages patient to tell the story of the problem(s) in own words | 0 (0) | 27(90) | 3(10) |  |
| 9 | Uses open and closed questioning technique, appropriately moving from open to closed | 0 (0) | 26(86.67) | 3(13.33) |  |
| 10 | Listens attentively, allowing patient to complete statements without interruption and leaving space for patient | 0 (0) | 27(90) | 3(10) |  |
| 11 | Facilitates patient's responses verbally and non–verbally | 0 (0) | 28 (93.33) | 2 (6.67) |  |
| 12 | Picks up verbal and non–verbal cues; checks out and acknowledges as appropriate | 0 (0) | 28 (93.33) | 2 (6.67) |  |
| 13 | Clarifies patient’s statements that are unclear or need amplification | 0 (0) | 17(56.67) | 13(43.33) |  |
| 14 | Periodically summarizes, invites the patient to correct the interpretation or provide further information | 0 (0) | 6 (20) | 24 (80) |  |
| 15 | Uses easily understood language, avoids jargon | 0 (0) | 0 (0) | 30 (100) |  |
| 16 | Establishes dates and sequence of events | 0 (0) | 30 (100) | 0 (0) |  |
|  | Overall score for Exploration of patient’s problems | Mean: 6.3 SD: 1.78 min 1 max 8 | | | |
| 17 | Actively determines and appropriately explores: patient’s ideas, concerns, expectations and effects about the problem | 0 (0) | 3(10) | 27(90) |  |
| 18 | Encourages patient to express feelings | 0 (0) | 17(56.67) | 13(43.33) |  |
| Overall scores for additional skills for  understanding the patient’s perspective | | Mean: 0.67 SD: 0.48 min 0 max 1 | | | |
| PROVIDING STRUCTURE | | | | | |
| 19 | Summarizes at the end of a specific line of inquiry to confirm understanding before moving on to the next section | 0 (0) | 0 (0) | 30 (100) |  |
| 20 | Progresses from one section to another using signposting; includes rationale for next section | 0 (0) | 0 (0) | 30 (100) |  |
| 21 | Structures interview in logical sequence | 0 (0) | 0 (0) | 30 (100) |  |
| 22 | Attends to timing and keeping interview on task | 0 (0) | 25 (16.67) | 5 (16.67) |  |
| Overall score for providing structure | | Mean: 0.83 SD: 0.38 min 0 max 1 | | | |
| BUILDING RELATIONSHIP | | | | | |
| 23 | Demonstrates appropriate non–verbal behavior | 0 (0) | 30 (100) | 0 (0) |  |
| 24 | If reads, writes notes or uses computer, does in a manner that does not interfere with dialogue or rapport | 0 (0) | 11 (36.67) | 19 (63.33) |  |
| 25 | Demonstrates appropriate confidence | 0 (0) | 29 (96.67) | 1 (3.33) |  |
|  | Overall score for Using appropriate non-verbal behavior | Mean: 2.33 SD: 0.55 min 1 max 3 | | | |
| 26 | Accepts legitimacy of patient’s views and feelings; is not judgmental | 0 (0) | 27(90) | 3(10) |  |
| 27 | Uses empathy to communicate understanding and appreciation of the patient’s feelings | 0 (0) | 28 (93.33) | 2 (6.67) |  |
| 28 | Provides support: expresses concern, understanding, willingness to help | 0 (0) | 24 (80.00) | 6(20) |  |
| 29 | Deals sensitively with embarrassing and disturbing topics and physical pain, including when associated with physical examination | 0 (0) | 1 (3.33) | 29 (96.67) |  |
|  | Overall score for Developing rapport | Mean: 2.67 SD: 0.75 min 0 max 3 | | | |
| 30 | Shares thinking with patient to encourage patient’s involvement | 0 (0) | 0 (0) | 30 (100) |  |
| 31 | Explains rationale for questions or parts of physical examination that could appear to be non-sequiturs | 0 (0) | 0 (0) | 30 (100) |  |
| 32 | During physical examination, explains process, asks permission | 0 (0) | 0 (0) | 30 (100) |  |
|  | Overall score for involving the patient | Mean: 0 SD: 0 min 0 max 0 | | | |
| CLOSING THE SESSION (Preliminary Explanation & Planning) | | | | | |
| 33 | Gives any preliminary information in clear well organized manner, avoids or explains jargon | 0 (0) | 12(40) | 18(60) |  |
| 34 | Checks patient understanding and acceptance of explanation and plans; ensures that concerns have been addressed | 0 (0) | 5(16.67) | 25(83.33) |  |
| 35 | Encourages patient to discuss any additional points and provides opportunity to do so | 0 (0) | 0 (0) | 30 (100) |  |
| 36 | Summarizes session briefly | 0 (0) | 0 (0) | 30 (100) |  |
| 37 | Contracts with patient re next steps for patient and physician | 0 (0) | 9(30) | 21(70) |  |
|  | Overall score for closing the session | Mean: 0.867 SD: 1.07 min 0 max 3 | | | |
